# LncRNA SNHG14 potentiates pancreatic cancer progression via modulation of annexin A2 expression by acting as a competing endogenous RNA for miR‐613

**DOI:** 10.1111/jcmm.14467

**Published:** 2019-09-12

**Authors:** Peng‐cheng Deng, Wei‐bo Chen, Hui‐hua Cai, Yong An, Xin‐quan Wu, Xue‐min Chen, Dong‐lin Sun, Yu Yang, Long‐qing Shi, Yong Yang

**Affiliations:** ^1^ Department of Hepatobiliary Surgery The First People's Hospital of Changzhou, The Third Hospital Affiliated to Soochow University Changzhou City China

**Keywords:** annexin A2, cell invasion, cell proliferation, miR‐613, pancreatic cancer, SNHG14

## Abstract

This study aimed to determine long non‐coding RNA (lncRNA) small nucleolar RNA host gene 14 (SNHG14) expression in pancreatic cancer and to explore the potential molecular actions of SNHG14 in mediating pancreatic cancer progression. Gene expression was detected by quantitative real‐time PCR. Cell proliferation, growth and invasion were detected by respective CCK‐8, colony formation, and transwell invasion assays. Protein levels were measured by Western blotting. Cell apoptosis and caspase‐3 activity were detected by flow cytometry and caspase‐3 activity assay. The link between miR‐613 and its targets was evaluated by luciferase reporter assay. In vivo tumour growth was evaluated using a xenograft model of nude mice. SNHG14 expression was up‐regulated in cancerous tissues from pancreatic cancer patients. High expression of SNHG14 was associated with poor tumour differentiation, advanced TNM stage and nodal metastasis. SNHG14 overexpression enhanced cell proliferative, growth and invasive abilities, and suppressed apoptotic rates and caspase‐3 activity in pancreatic cancer cells, while SNHG14 knockdown exerted opposite effects. Mechanistic studies revealed that miR‐613 was targeted by SNHG14, and Annexin A2 (ANXA2) was targeted and inversely regulated by miR‐613 in pancreatic cancer cells. In vivo studies showed that SNHG14 knockdown attenuated tumour growth. MiR‐613 was down‐regulated and ANXA2 was up‐regulated in the pancreatic cancer tissues, and SNHG14 expression levels were inversely correlated with miR‐613 expression levels and positively correlated with the ANXA2 mRNA expression levels. Collectively, our results suggest that SNHG14 potentiates pancreatic cancer progression through modulation of annexin A2 expression via acting as a competing endogenous RNA for miR‐613.

## 1 INTRODUCTION

Despite advancements in cancer research, pancreatic cancer remains one of the severe diseases nowadays. In China, the health burden from this cancer is continuing to rise, especially in rural areas. In fact, China accounted for about 20% of all new pancreatic cancer cases in the world.^1^, ^2^ Surgical resection and chemotherapy are the major therapeutic strategies for pancreatic cancer.^3^ Unfortunately, drug resistance is still a major obstacle for current regimens. As a result, there has been no obvious improvement in the life expectancy of patients over the last decades, and survival rates for 5 years of the patients remains lower than 5%.^4^, ^5^ In this regard, a thorough understanding about the detailed mechanisms underlying pancreatic cancer progression is urgently needed to aid in the development of novel therapeutic targets.

Recently, growing evidence has implied that non‐coding RNAs (ncRNAs) were important regulators in cellular biology and pathological processes. MicroRNAs (miRNAs) are a class of small ncRNAs with about 22 nucleotides in length and can bind to the 3′‐untranslated region (3′ UTR) of the genes and repress the expression of specific genes.^6^ Long ncRNAs (lncRNAs) are transcripts with more than 200 nucleotides in length that function like sponges and bind to miRNAs in a competitive manner. Numerous lncRNAs have identified for their aberrant expression profiles in tumours.^7^, ^8^, ^9^ Dysregulation of lncRNAs may promote tumour cell proliferation, invasion and metastasis, and lncRNAs can exert tumour enhancing or suppressive effects.^10^ One important molecular mechanism of lncRNA underlying cancer progression is that lncRNA acts as competing endogenous RNA (ceRNA) to repress its targeted miRNA expression, thereby regulating specific genes targeted by miRNAs.^11^ In addition, various studies have implied that lncRNAs could serve as diagnostic biomarkers or novel therapeutic targets for cancers.^11^

In the current investigations, the role of the lncRNA small nucleolar RNA host gene 14 (SNHG14) in pancreatic cancer was explored and found that up‐regulation of SNHG14 was detected in tissues and cell lines of the pancreatic cancer. The functional assays showed that SNHG14 promoted pancreatic cancer cell proliferative, growth and invasive abilities and reduced cell apoptotic rates and caspase‐3 activity. We also found that SNGH14 modulated annexin A2 (ANXA2) expression via targeting miR‐613 in pancreatic cancer cells. In addition, in vivo studies indicated that SNHG14 knockdown exerted anti‐tumour effects. Our studies suggested that SNHG14 may be a prospective target for the management of pancreatic cancer.

## 2 MATERIALS AND METHODS

### 2.1 Collection of clinical samples

Pancreatic cancer tissues and the normal pancreatic tissues adjacent to the tumour were isolated from 45 patients who have received surgical resection at the First People's Hospital of Changzhou. All the collected tissue samples were stored at –80°C for further experimental assays. All cases were histologically confirmed as pancreatic cancer by three independent histopathologists. The study was under the approval of Ethics Committee of the First People's Hospital of Changzhou, and each patient signed the written informed consent for the collection of tissue samples.

### 2.2 Cell culture, oligonucleotides and cell transfections

The normal immortalized human pancreatic epithelial cell line (HPDE6C7) and four human pancreatic cancer cell lines (CFPAC‐1, BXPC3, L3.6pl and Panc‐1) were purchased from the Cell Bank of the Chinese Academy of Sciences (Shanghai, China). All the relevant cells were cultured in DMEM (Sigma‐Aldrich, St. Louis) supplemented with 10% fetal bovine serum (FBS; HyClone, Logan, USA) and kept in a humidified incubator with 5% carbon dioxide at 37°C. The pcDNA3.1‐SNHG14 and pcDNA3.1‐ANXA2 (vector with SNHG4 or ANXA2 overexpression) and empty vectors were from GenePharma (Shanghai, China). The miR‐613 mimic and its negative control (NC), and SNHG14 small interfering RNA (siRNA) (si‐SNHG14) and scrambled siRNA for SNHG14 (si‐NC) were from RiboBio (Guangzhou, China). For cell transfections, cells were grown on 6‐well plates until 60% confluence, and then cells were transfected by miRNA, siRNA, or plasmid using Lipofectamine 2000 reagent (Invitrogen, Carlsbad).

### 2.3 Gene expression levels as detected by quantitative real‐time PCR (qRT‐PCR) analysis

Total RNA isolation from tissues and cells was performed using TRIzol reagent (Invitrogen). LncRNA SNHG14 and ANXA2 mRNA levels were detected by real‐qRT‐PCR using a SYBR Premix ExTaq Reverse Transcription PCR kit (Takara, Dalian, China). GAPDH was used to normalize SNHG14 and ANXA2 mRNA expression levels. For miRNA detection, miR‐613 level was detected by qRT‐PCR using TaqMan assay kit (Applied Biosystems, Foster City, CA) and U6 was used as to normalize miR‐613 expression level. SNHG14, ANXA2 and miR‐613 expression levels were calculated using the 2^–∆∆Ct^ formula.

### 2.4 Cell proliferative ability as detected by CCK‐8 assay

Cells (1 × 10^4^ cells) after being transfected with oligonucleotides were seeded onto a 96‐well plate. Cell proliferative ability was detected using a Cell Counting Kit‐8 (CCK‐8) kit (Dojindo, Kumamoto, Japan) according to the manuals from manufacturer. The optical density value was determined at 0, 24, 48 and 72 hours at a wavelength of 450 nm.

### 2.5 Cell growth as detected by colony formation assay

Cells after being transfected with oligonucleotides were seeded onto a 6‐cm culture dish. Fourteen days post‐culture, cells were fixed by 70% ethanol for a duration of 10 minutes. After that, the fixed cells were stained by 0.1% crystal violet for a duration of 30 minutes. Visible colonies from randomly selected five fields were manually counted under a microscope (LEICA, Wetzlar, Germany).

### 2.6 Cell invasive abilities as detected by Transwell invasion assay

For invasion assay, cells after being transfected with oligonucleotides were seeded onto the upper chamber of the transwell with Matrigel‐coated membrane (8‐µm pore size; Corning Inc, Corning, USA), and DMEM supplemented with 10% FBS were served as chemoattractant and were added into the lower chamber. After a further incubation for 24 hours, the invaded cells on the membrane were stained by 0.1% crystal violet for the duration of 30 minutes and quantified by randomly selecting five fields under a microscope (LEICA).

### 2.7 Cell apoptotic rates as detected by flow cytometry

Cells after being transfected with oligonucleotides were trypsinized and fixed by ice‐cold 70% ethanol for the duration of 30 minutes. After that, the transfected cells were incubated with 20 mg/mL RNase (Sigma‐Aldrich) at 37°C for the duration of 1 hour. For cell apoptosis analysis, cells were stained with FITC Annexin V and propidium iodide (Beyotime, Beijing, China), and then apoptotic cells were detected using a FACSCalibur flow cytometer (BD Biosciences, Franklin Lakes, NJ).

### 2.8 Caspase‐3 activity

Cells after being transfected with oligonucleotides were subjected to the capase‐3 activity determination using a Caspase‐3 activity assay kit (Abcam, Cambridge, UK) according to the manufacturer's protocol.

### 2.9 Luciferase reporter assay

For luciferase reporter assay, pLG3 vector (Promega, Madison, WI) was used to construct the luciferase reporter vectors. Panc‐1 cells were seeded onto a 96‐well plate with each well containing 1 × 10^4^ cells. After reaching 60% confluence, the cells were co‐transfected with pGL3‐ANXA2 3′‐UTR plasmid (wild type), pGL3‐ANXA2 3′‐UTR plasmid (mutated), pGL3‐SNHG14 (wild type) or pGL3‐SNHG14 (mutated) and miR‐613 mimics or control miRNA using Lipofectamine 2000 reagent (Invitrogen). At 48 hours following transfection, luciferase activity of different vector constructs was detected by the Dual‐Luciferase Reporter Assay System (Promega). The relative luciferase activity was expressed as the ratio between firefly and Renilla luciferase activities.

### 2.10 RNA immunoprecipitation

Panc‐1 cells were co‐transfected with pSL‐MS2, pSL‐MS2‐SNHG14 or pSL‐MS2‐SNHG14 (MUT) along with pMS2‐GFP (Addgene). At 48 h after co‐transfection, cells were processed to perform RNA immunoprecipitation (RIP) experiments using a GFP antibody (Roche, Basel, Switzerland) and the Magna RIP^TM^ RNA‐Binding Protein Immunoprecipitation Kit (Millipore, Burlington, USA) according to the manufacturer's protocol. The RNA fraction was then purified and analysed by qRT‐PCR.

### 2.11 Fluorescent in situ hybridization

The fluorescent in situ hybridization (FISH) assay was performed to detect the localization of SNHG14 in Panc‐1 cells. The RNA probes for SNHG14 were designed and synthesized by RiboBio (Guangzhou, China). Briefly, cells were fixed with 4% paraformaldehyde (Sigma‐Aldrich) at room temperature for 15 minutes and the cells were then treated with pepsin and dehydrated with graded ethanol. After that, cells were then subjected to incubation with FISH probe in the hybridization buffer, followed by staining with 4′ 6‐diamidino‐2‐phenylindole (DAPI) for 5 minutes. The fluorescent signals were determined under a fluorescent microscope (Olympus BX51, Tokyo, Japan).

### 2.12 Western blotting

Proteins extraction from cells and tissues was performed using RIPA buffer, and then the extracted proteins separated by gel electrophoresis with a 10% SDS‐PAGE gel. Separated proteins were then transferred onto polyvinylidene difluoride membranes. The membranes were then incubated with 5% skimmed milk for a duration of 2 hours at room temperature with agitation, after that, the membranes were incubated with ANXA2 primary antibody (1:1000; ab41803; Abcam) or β‐actin primary antibody (1:2000; ab8227, Abcam) at 4°C overnight, followed by incubating with relevant secondary antibodies with horse‐radish peroxidase conjugation (1:2000; Santa Cruz Biotechnology, Dallas) for 2 hours at room temperature. Western blot bands were detected using an enhanced chemiluminescence kit (Pierce, Rockford, USA).

### 2.13 In vivo xenograft model

All animal experimental procedures were under the approval of the Animal Research Committee of the First People's Hospital of Changzhou. Twelve 4‐week‐old BALB/c nude mice were randomly divided into two groups. Panc‐1 cells transfected with si‐NC or si‐SNHG14 (2 × 10^6^ cells/mice) were administered into the neck area of the nude mice by subcutaneous injections. The tumour volume of the mice was monitored up to 42 days with measurement at a 7‐day interval. The tumour volume was calculated using the following formula: volume = (width × length × height)/2. The tumour tissues were also dissected from the sacrificed mice for further experimental assays.

### 2.14 Statistical analysis

All results are expressed as the mean ± standard deviation. The graph plots and statistics were performed using GraphPad Prism software (version 6.0; GraphPad Software, La Jolla, CA). Statistical significance was evaluated by Student's *t* test or analysis of variance followed by multiple comparison tests. Chi‐square test used to calculate *P* values for the categorical data. Pearson's correlation analysis determined the correlation between two variables. Differences were considered statistically significant when *P* < 0.05. All results are representative of at least three independent experiments.

## 3 RESULTS

### 3.1 Up‐regulation of SNHG14 is detected in in pancreatic cancer and SNHG14 is correlated with the clinical features of pancreatic cancer patients

**Figure 1:**
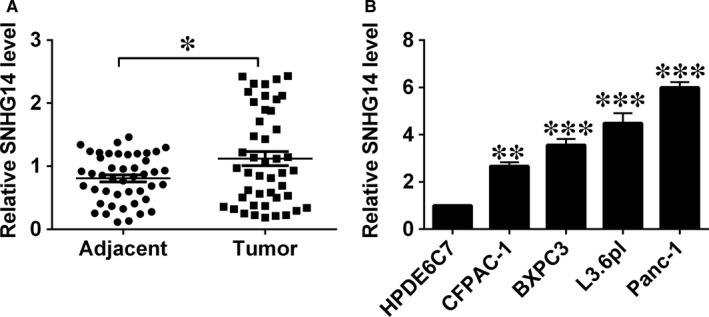
SNHG14 was up‐regulated in pancreatic cancer tissues and cancer cells. (A) qRT‐PCR analysis of SNHG14 expression levels in adjacent normal pancreatic tissues and pancreatic cancer tissues from patients with pancreatic cancer. (B) qRT‐PCR analysis of SNHG14 expression levels in normal pancreatic cell line (HPDE6C7) and pancreatic cancer cell lines (CFPAC‐1, BXPC3, L3.6pl and Panc‐1). N = 3, significant differences compared to respective controls were shown as *P < 0.05, **P < 0.01 and ***P < 0.001

The qRT‐PCR results detected the up‐regulation of SNHG14 in pancreatic cancer tissues, with the relatively higher SNHG14 expression level than that in adjacent non‐tumour tissues (Figure 1A). Similarly, up‐regulation of SNHG14 was also detected in the pancreatic cancer cell lines (BXPC3, CFPAC‐1 L3.6pl and Panc‐1) when compared with that in the normal cell line (HPDE6C7; Figure 1B). Low or high expression of SNHG14 in cancerous tissues from pancreatic cancer patients was classified based on the median values. High SNHG14 expression in the cancerous tissues showed a positive correlation with poorer tumour differentiation, more advanced TNM stage and nodal metastasis (Table 1), but had no associated with age, sex and tumour size in patients with pancreatic cancer (Table 1).

**Table 1:** The correlation between SNGH14 expression level and clinical characteristics of pancreatic cancer patients

Clinical parameters	SNHG14 expression		*P* value
	Low expression (N = 22)	High expression (N = 23)	
Age			
<60 years old	10	13	0.4578
≥60 years old	12	10	
Gender			
Male	13	12	0.6047
Female	9	11	
Tumour differentiation			
Well/moderate	14	7	**0.0256**
Poor	8	16	
TNG stage			
I‐II	15	7	**0.0113**
III‐IV	7	16	
Tumour size			
<2 cm	14	9	0.1002
≥2 cm	8	14	
Nodal metastasis			
No	15	8	**0.0251**
Yes	7	15	

*P* < 0.05 is considered statistically signficant (in bold).

### 3.2 Up‐regulation of SNHG14 promoted pancreatic cancer cell proliferative, growth and invasive potentials, and reduced apoptotic rates and caspase‐3 activity

**Figure 2:**
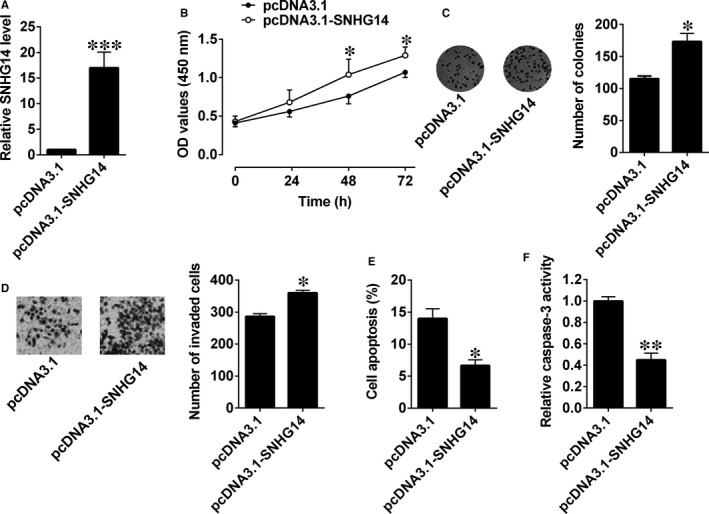
Up‐regulation of SNHG14 promoted cell proliferation, cell growth and cell invasion, and suppressed cell apoptosis in pancreatic cancer cells. (A) qRT‐PCR analysis of SNHG14 expression levels in L3.6pl cells after pcDNA3.1 or pcDNA3.1‐SNHG14 transfection. (B) Cell proliferation of L3.6pl cells after pcDNA3.1 or pcDNA3.1‐SNHG14 transfection was determined by CCK‐8 assay. (C) Cell growth, (D) cell invasion, (E) cell apoptosis and (F) caspase‐3 activity of L3.6pl cells after pcDNA3.1 or pcDNA3.1‐SNHG14 transfection were measured by colony formation assay, Transwell invasion assay, flow cytometry and caspase‐3 activity assay kit respectively. N = 3, significant differences compared to respective controls were shown as *P < 0.05 and ***P < 0.001

We then constructed an SNHG14‐overexpressing vector (pcDNA3.1‐SNHG14) and used an empty vector as the NC (pcDNA3.1). SNHG14 expression levels in L3.6pl cells after being transfected with pcDNA3.1‐SNHG14 were approximately 16‐fold higher than control group (Figure 2A). CCK‐8 assay revealed that cell proliferation was enhanced in L3.6pl cells with pcDNA3.1‐SNHG14 transfection compared with that in cells with empty vector transfection (Figure 2B). Consistently, transfection with pcDNA3.1‐SNHG14 significantly increased growth of pancreatic cancer cells (Figure 2C). Cell invasion assay showed that the invasive potentials of cancer cells in the SNHG14 group was markedly potentiated when compared with the control group (Figure 2D). Also, cell apoptotic rates and caspase‐3 activity were significantly reduced after pcDNA3.1‐SNHG14 transfection in L3.6pl cells (Figure 2E,F).

### 3.3 Down‐regulation of SNHG14 suppressed pancreatic cancer cell proliferative, growth, and invasive potentials and increased cell apoptotic rates and caspase‐3 activity

**Figure 3:**
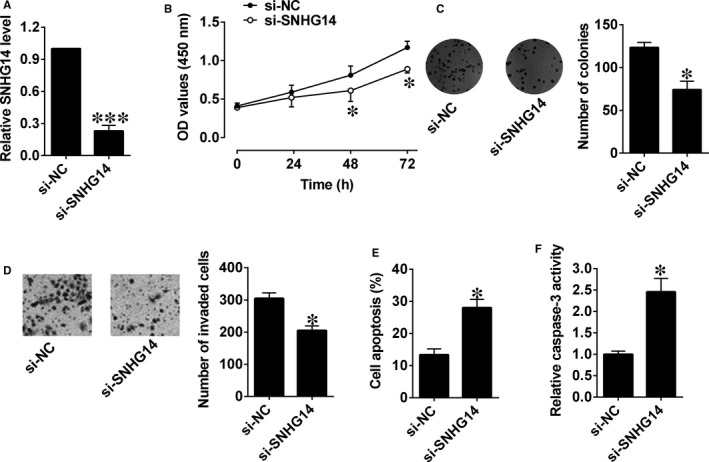
Down‐regulation of SNHG14 suppressed cell proliferation, cell growth and cell invasion, and induced cell apoptosis in pancreatic cancer cells. (A) qRT‐PCR analysis of SNHG14 expression levels in Panc‐1 cells after si‐NC or si‐SNHG14 transfection. (B) Cell proliferation of Panc‐1 cells after si‐NC or si‐SNHG14 transfection was determined by CCK‐8 assay. (C) Cell growth, (D) cell invasion, (E) cell apoptosis and (F) caspase‐3 activity of Panc‐1 cells after si‐NC or si‐SNHG14 transfection were measured by colony formation assay, Transwell invasion assay, and flow cytometry and caspase‐3 activity assay kit, respectively. N = 3, significant differences compared to respective controls were shown as *P < 0.05 and ***P < 0.001

Similarly, silencing of SNHG14 in Panc‐1 cells was carried out by transfecting with si‐SNHG14, which markedly decreased the expression level of SNHG14 in Panc‐1 cells (Figure 3A). The cell proliferative potential and growth were markedly suppressed in Panc‐1 cells after being transfected with si‐SNHG14 compared to control group (Figure 3B,C). Moreover, the invasive ability of cells was inhibited and cell apoptosis and caspase‐3 activity were obviously increased after si‐SNHG14 transfection (Figure 3D‐F).

### 3.4 SNHG14 down‐regulated miR‐613 expression in pancreatic cancer cells

**Figure 4:**
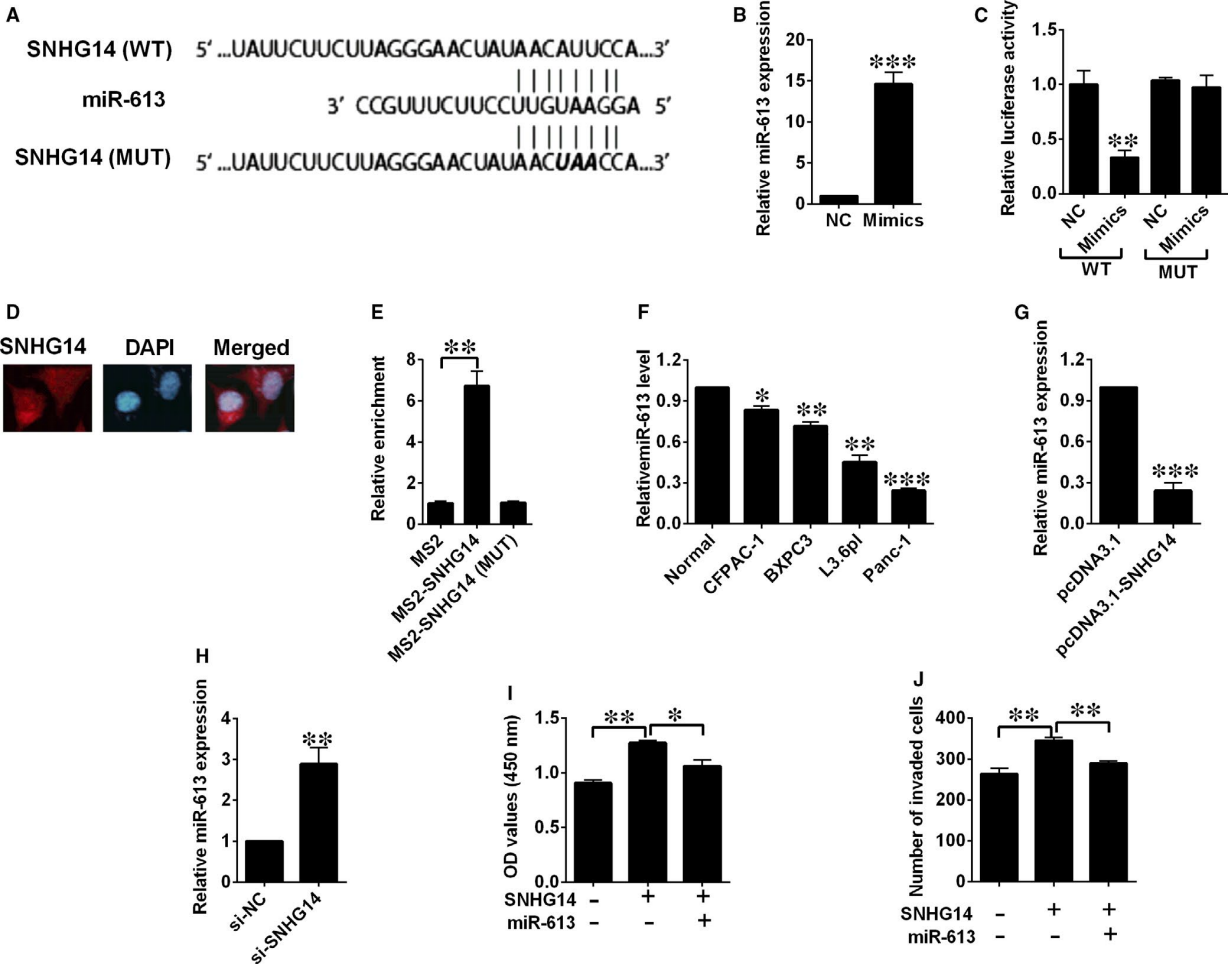
SNHG14 down‐regulated the expression of miR‐613 in pancreatic cancer cells. (A) Putative miR‐613 binding sequences of SNHG14 and the reporter constructs showing the wild type (WT) SNHG14 sequence and the mutant (MUT) SNHG14 sequence. (B) qRT‐PCR analysis of miR‐613 expression levels in Panc‐1 cells after scrambled miRNA (NC) or miR‐613 mimics transfection. (C) miR‐613 mimics transfection suppressed the luciferase activity of the WT but not the MUT SNHG14 reporter in Panc‐1 cells. (D) Detection of SNHG14 using FISH assay in Panc‐1 cells. (E) MS‐RIP followed by qRT‐PCR to detect the interaction between SNHG14 and miR‐613 in Panc‐1 cells. (F) qRT‐PCR analysis of miR‐613 expression levels in normal pancreatic cell line and pancreatic cancer cell lines. (G) qRT‐PCR analysis of miR‐613 expression levels in Panc‐1 cells after pcDNA3.1 or pcDNA3.1‐SNHG14 transfection. (H) qRT‐PCR analysis of miR‐613 expression levels in Panc‐1 cells after si‐NC or si‐SNHG14 transfection. (I) Cell proliferation and (J) cell invasion of Panc‐1 cells co‐transfected with pcDNA3.1 + scrambled miRNA, pcDNA3.1‐SNHG14 + scrambled miRNA, or pcDNA3.1‐SNHG14 + miR‐613 mimics were determined by CCK‐8 assay and Transwell invasion assay, respectively. N = 3, significant differences compared to respective controls were shown as *P < 0.05, **P < 0.01 and ***P < 0.001

We predicted potential targets of SNHG14 using online software (DIANA Tools), and miR‐613 was found to be one of the potential targets. As tumour‐suppressive effects of miR‐613 was highlighted in our previous study for pancreatic cancer,^12^ we selected miR‐613 to examine the potential relationship between SNHG14 and miR‐613 using luciferase reporter assay. Luciferase reporter plasmids with fragments of SNHG14 (wild type or mutated) were constructed (Figure 4A). On the other hand, transfection by miR‐613 mimics successfully up‐regulated miR‐613 expression (Figure 4B). Co‐transfection with miR‐613 mimics and reporter vectors with fragment of SNHG14 (wild type) markedly attenuated luciferase activity in Panc‐1 cells (Figure 4C), while co‐transfection with miR‐613 mimics and reporter vectors with the mutated fragment of SNHG14 failed to attenuate the luciferase activity (Figure 4C). The FISH results showed that SNHG14 was mainly detected in the cytoplasm of Panc‐1 cells (Figure 4D). More importantly, the RIP assay showed that SNHG14 RIP was significantly enriched for miR‐613 in Panc‐1 cells compared with MS2 and the mutant vector (Figure 4E). Furthermore, down‐regulation of miR‐613 was detected in the pancreatic cancer cell lines when compared with the normal cell line (Figure 4F). Ectopic expression of SNHG14 by pcDNA3.1‐SNHG14 transfection significantly decreased miR‐613 expression level, while silencing of SNHG14 by si‐SNHG14 transfection up‐regulated miR‐613 expression (Figure 4G,H). In addition, CCK‐8 and transwell invasion assays revealed that miR‐613 overexpression partially restored the increased proliferative and invasive abilities of Panc‐1 cells induced by pcDNA3.1‐SNHG14 transfection (Figure 4I,J).

### 3.5 ANXA2 is targeted by miR‐613 in pancreatic cancer cells

**Figure 5:**
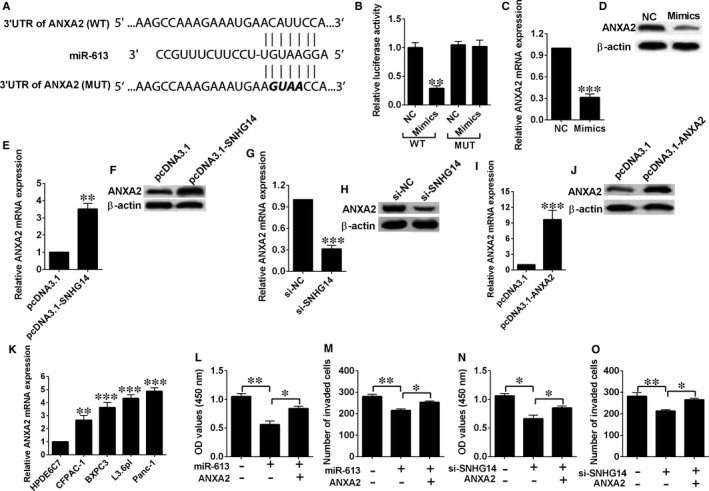
ANXA2 was a downstream target of miR‐613 in pancreatic cancer cells. (A) Putative miR‐613 binding sequences in the 3′UTR of ANXA2 and the reporter constructs showing the wild type (WT) ANXA2 3′UTR sequence and the mutant (MUT) ANXA2 3′UTR sequence. (B) miR‐613 mimics transfection suppressed the luciferase activity of the WT but not the MUT ANXA2 3’UTR reporter in Panc‐1 cells. (C) qRT‐PCR analysis and (D) Western blot analysis of ANXA2 mRNA and protein expression levels in Panc‐1 cells after scrambled miRNA (NC) or miR‐613 mimics transfection. (E) qRT‐PCR analysis and (F) Western blot analysis of ANXA2 mRNA and protein expression levels in Panc‐1 cells after pcDNA3.1 or pcDNA3.1‐SNHG14 transfection. (G) qRT‐PCR analysis and (H) Western blot analysis of ANXA2 mRNA and protein expression levels in Panc‐1 cells after si‐NC or si‐SNHG14 transfection. (I) qRT‐PCR analysis and (J) Western blot analysis of ANXA2 mRNA and protein expression levels in Panc‐1 cells after pcDNA3.1 or pcDNA3.1‐ANXA2 transfection. (K) qRT‐PCR analysis of ANXA2 mRNA expression levels in normal pancreatic cell line (HPDE6C7) and pancreatic cancer cell lines (CFPAC‐1, BXPC3, L3.6pl and Panc‐1). (L) Cell proliferation and (M) cell invasion of Panc‐1 cells co‐transfected with pcDNA3.1 + scrambled miRNA, pcDNA3.1 + scrambled miRNA, or pcDNA3.1‐ANXA2 + miR‐613 mimics were determined by CCK‐8 assay and Transwell invasion assay, respectively. (N) Cell proliferation and (O) cell invasion of Panc‐1 cells co‐transfected with si‐NC + pcDNA3.1, si‐SNHG14 + pcDNA3.1, si‐SNHG14 + pcDNA3.1‐ANXA2 were determined by CCK‐8 assay and Trasnwell invasion assay, respectively. N = 3, significant differences compared to respective controls were shown as *P < 0.05, **P < 0.01 and ***P < 0.001

We also predicted target genes of miR‐613 using TargetScan, and among the predicted targets of miR‐613, ANXA2 was selected for further investigation in our study due its well‐known role in cancer progression.^13^ In a similar manner, luciferase reporter assay was performed to confirm this interaction. Reporter plasmids containing the wild‐type or mutated 3′‐UTR of ANXA2 were constructed (Figure 5A). Co‐transfection with miR‐613 mimics and reporter vectors with the ANXA2 3′‐UTR (wild type) inhibited luciferase activity, while co‐transfection with miR‐613 mimics and reporter plasmids with mutated ANXA2 3′‐UTR failed to change luciferase activity in Panc‐1 cells (Figure 5B). Ectopic expression of miR‐613 suppressed the expression of ANXA2 mRNA and protein in Panc‐1 cells (Figure 5C and 5D). SNHG14 overexpression up‐regulated ANXA2 expression, while SNHG14 silence down‐regulated ANXA2 expression (Figure 5E‐H). In addition, pcDNA3.1‐ANXA2 transfection markedly elevated the expression levels of ANXA2 mRNA and protein (Figure 5I,J), and the expression of ANXA2 was significantly up‐regulated in pancreatic cancer cells (Figure 5K). Moreover, ectopic expression of ANXA2 restored the decreased cell proliferation and invasion abilities induced by miR‐613 mimics transfection or SNHG14 knockdown in Panc‐1 cells (Figure 5L‐O).

### 3.6 Knockdown of SNHG14 suppressed in vivo xenograft tumour growth

**Figure 6:**
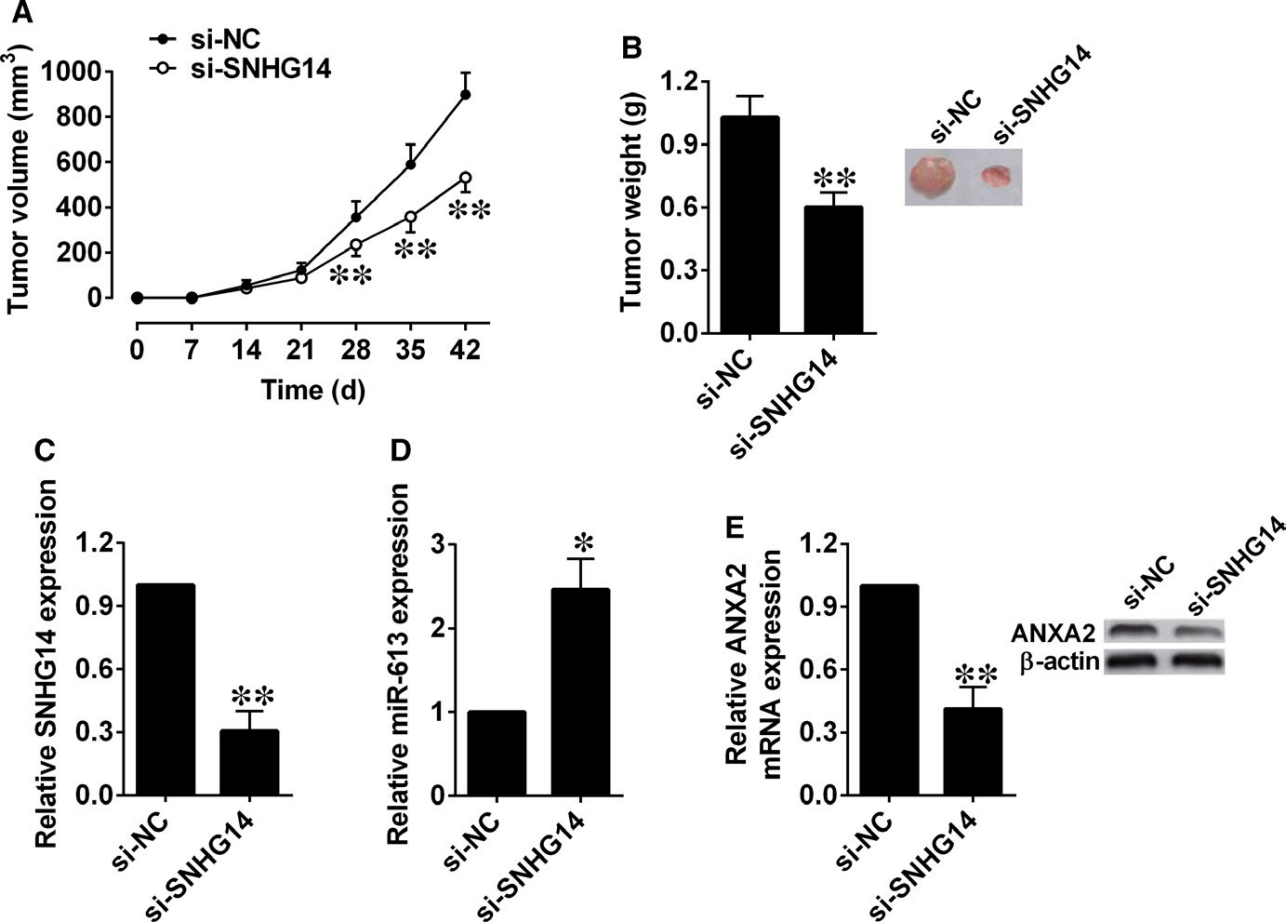
Knockdown of SNHG14 suppressed xenograft tumour growth in vivo. (A) Tumour volume and (B) tumour weight changes in the mice bearing Panc‐1 cells with si‐NC or si‐SNHG14 transfection. (C) qRT‐PCR analysis of SNHG14 expression levels in the isolated tumour tissues. (D) qRT‐PCR analysis of miR‐613 expression levels in the isolated tumour tissues. (E) qRT‐PCR and Western blot analysis of ANXA2 mRNA and protein expression levels in the isolated tumour tissues. N = 6, significant differences compared to respective controls were shown as *P < 0.05 and **P < 0.01

The nude mice were injected subcutaneously with Panc‐1 cells after being transfected with si‐NC or si‐SNHG14. In the SNHG14 knockdown group, reduced tumour volume was observed at 28, 35 and 42 days after cell injections compared (Figure 6A). After dissection, tumour weight was measured, and a lower tumour weight was observed in the SNHG14 knockdown group than the si‐NC group (Figure 6B). SNHG14 and miR‐613 expression levels in the excised tumours were assessed by qRT‐PCR. SNHG14 expression was down‐regulated in the si‐SNHG14 group when compared with si‐NC group (Figure 6C), while miR‐613 expression was up‐regulated in the si‐SNHG14 group compared with si‐NC group (Figure 6D). Moreover, ANXA2 mRNA and protein levels were also examined, and ANXA2 expression was found to be down‐regulated in the si‐SNHG14 group (Figure 6E).

### 3.7 MiR‐613 was down‐regulated and ANXA2 was up‐regulated in cancerous tissues from pancreatic cancer patients

**Figure 7:**
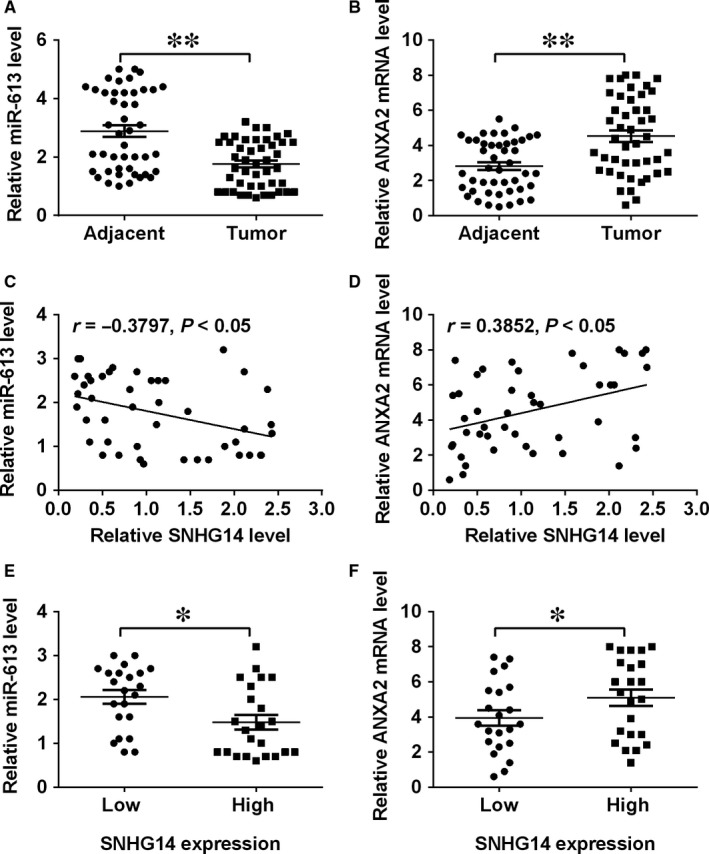
MiR‐613 was down‐regulated and ANXA2 was up‐regulated in pancreatic cancer tissues. (A) qRT‐PCR analysis of miR‐613 expression levels in adjacent normal pancreatic tissues and pancreatic cancer tissues from patients with pancreatic cancer. (B) qRT‐PCR analysis of ANXA2 mRNA expression levels in adjacent normal pancreatic tissues and pancreatic cancer tissues from patients with pancreatic cancer. (C) The correlation between SNHG14 expression level and miR‐613 expression level in pancreatic cancer tissues was analysed by Pearson's correlation analysis. (D) The correlation between SNHG14 expression level and ANXA2 mRNA expression level in pancreatic cancer tissues was analysed by Pearson's correlation analysis. (E) qRT‐PCR analysis of miR‐613 expressin levels in pancreatic cancer tissues with low and high expression of SNHG14. (F) qRT‐CPR analysis of ANXA2 mRNA expression levels in pancreatic cancer tissues with low and high expression of SNHG14. Significant differences compared to respective controls were shown as *P < 0.05 and **P < 0.01

Results from the qRT‐PCR assay found that that miR‐613 expression levels were decreased, while ANXA2 mRNA expression levels were increased in cancerous tissues when compared with adjacent normal pancreatic tissues (Figure 7A,B). Further correlation analysis showed that SNHG14 expression levels were inversely correlated with miR‐613 expression levels and positively correlated with those of ANXA2 in pancreatic cancer tissues (Figure 7C,D). In addition, the miR‐613 expression level was lower and the ANXA2 mRNA expression level was higher in the pancreatic cancer tissues with high expression of SNHG14 than those with low expression of SNHG14 (Figure 7E,F).

## 4 DISCUSSION

A large number of lncRNAs have been found to participate in the pancreatic cancer progression. For instance, the lncRNA PANDAR is highly up‐regulated in the tissues resected from pancreatic ductal adenocarcinoma, and PANDAR is correlated with advanced tumour stage and vascular invasion.^14^ Up‐regulation of lncRNA AFAP1 antisense RNA 1 has also been detected in the cancerous tissues from pancreatic cancer patients, and its use as a prognostic marker has been suggested, as AFAPA1 antisense RNA1 overexpression predicted cancer metastasis and poor survival of pancreatic cancer patients.^15^ In addition, expression of the lncRNA plasmacytoma variant translocation 1 (PVT1) expression is enhanced in pancreatic cancer, and it was also identified as a positive modulator of gemcitabine sensitivity, as silencing of PVT1 sensitize the cancer cells to gemcitabine.^16^ However, the function and mechanism of lncRNAs in pancreatic cancer are remaining elusive.

In the current investigations, we observed that lncRNA SNHG14 was highly expressed in pancreatic tissues as well as cell lines. As for SNHG14, it is also known as UBE3A‐ATS and suppresses UBE3A expression.^17^ SNHG14 has been found to regulate microglial activation in cerebral infarction.^18^ In a previous cancer study, SNHG14 was shown to be overexpressed in gastric cancer tissues and cells.^19^ In clear cell renal cell carcinoma, SNHG14 promotes cell migratory and invasive abilities through up‐regulation of N‐WASP protein level.^20^ On the other hand, SNHG14 potentiates the chemoresistance of breast cancer to trastuzumab.^21^ The above findings indicate that SNHG14 may be oncogenic in pancreatic cancer. Mechanistically, SNGH14 promotes microglial activation by regulating phospholipase A2 group IVA signalling by sponging miR‐145‐5p in cerebral infarction.^18^ Liu et al, showed that SNHG14 enhances progression of gastric cancer via modulating miR‐145/ SRY‐box 9 signalling.^19^ In breast cancer, SNHG14 induces chemoresistance via regulating poly(A) binding protein cytoplasmic 1 expression through H3K26 acetylation.^21^ These results suggest that SNHG14 regulates distinct signalling pathways in a cancer‐type dependent manner.

Here, functional assays showed that up‐regulation of SNHG14 promoted proliferative, growth, and invasive abilities, and suppressed cell apoptosis in pancreatic cancer cells, while silencing of SNHG14 exerted the opposite effects. Furthermore, miR‐613 was experimentally confirmed as a target of SNHG14, and SNHG14 down‐regulated miR‐613 expression in pancreatic cancer cells. The key functions of miRNAs in pancreatic cancer progression have also been highlighted by various other studies. MiR‐130b has been shown to inhibit cell proliferative and invasive abilities in pancreatic cancer through targeting signal transducer and activator of transcription 3 and was identified as a prognostic marker.^22^ Down‐regulation of miR‐29 was detected in activated pancreatic stellate cells, which is related to increased deposition of extracellular matrix deposition in pancreatic cancer.^23^ miR‐320a also promotes pancreatic cancer progression, metastasis and contributes to the resistance to 5‐fluorouracil.^24^ In our previous study, miR‐613 expression was down‐regulated in pancreatic cancer tissues and cancer cell lines, which positively correlated with poor prognosis of pancreatic cancer patients. Additionally, miR‐613 inhibited cell proliferative, invasive and migratory abilities, as well as increased cell apoptotic rates and induced G0/G1 cell cycle arrest of pancreatic cancer cells. HOTAIR functioned as a ceRNA to down‐regulate miR‐613 expression, and miR‐613 regulated the expression of its downstream target Notch3.^12^ Similarly, enhanced expression of miR‐613 restored the increased cell proliferation and invasion abilities induced by SNHG14.

ANXA2 is a tumour‐associated protein and acts as an oncogene to potentiate cancer progression including ovarian cancer, hepatoma and breast cancer.^25^, ^26^ In addition, the ANX family of proteins are associated with both chemotherapy and radiotherapy resistance. A high ANXA2 expression has been associated with poor prognosis of pancreatic cancer patients.^27^ Thus, down‐regulation of ANXA2 may have prospective effects. This in vivo study showed that silencing of SNHG14 increased the miR‐613 expression level, which subsequently down‐regulated ANXA2 expression and suppressed tumour growth.

However, this study has several limitations that should be addressed. The link between SNHG14 expression and the overall survival of pancreatic cancer patients should be examined to further confirm the prognostic potential of SNHG14 in pancreatic cancer. Localization of SNHG14 should be determined by immunohistochemistry to confirm the up‐regulation of SNGH14 in pancreatic cancer tissues. In addition, because SNHG14 and miR‐613 have more than just one targeted gene, other potential targets of SNHG14 and miR‐613 may be investigated in future studies.

In conclusion, this study identified the interaction network of SNHG14 and miR‐613 in pancreatic cancer. Our current findings suggest that SNHG14 potentiates pancreatic cancer progression through modulation of annexin A2 expression via acting as a competing endogenous RNA for miR‐613, and that the SNHG14‐miR‐613‐ANXA2 axis may represent a key signalling pathway for pancreatic cancer progression.

## CONFLICT OF INTEREST

None.

## Data Availability

The datasets generated during this study are available.
